# Global trends and emerging hotspots in Sertoli cell research: A 24-year bibliometric analysis (2000–2024)

**DOI:** 10.1097/MD.0000000000050290

**Published:** 2026-09-11

**Authors:** Anan Yu, Hongyu Chen

**Affiliations:** a Hangzhou TCM Hospital of Zhejiang Chinese Medical University (Hangzhou Hospital of Traditional Chinese Medicine), Hangzhou, China.

**Keywords:** bibliometric analysis, male infertility, Sertoli cells, spermatogenesis, Web of Science

## Abstract

**Background:**

Sertoli cells play a pivotal role in spermatogenesis by supporting germ cell development and maintaining the blood–testis barrier. Understanding their functions is critical for addressing male infertility, a growing global health concern. This study aims to map the knowledge structure and research hotspots in Sertoli cell-related spermatogenesis through a bibliometric analysis.

**Methods:**

We retrieved literature from the Web of Science Core Collection (2000–2024) using keywords related to Sertoli cells and spermatogenesis. A total of 6268 articles were analyzed using bibliometric tools (CiteSpace, VOSviewer, R-Studio, and Scimago Graphica) to assess publication trends, collaborations, influential journals, and keyword evolution.

**Results:**

A total of 6268 relevant articles were included. The number of annual publications has remained relatively stable overall, from 211 articles in 2000 to 301 articles in 2024. The United States (1479 articles) and China (1324 articles) dominated research output. Leading institutions included the Population Council (USA) and Inserm (France). Core authors Cheng, C Yan (197 articles) and Mruk, Dolores D (115 articles) focused on Sertoli-germ cell interactions. The *Biology of Reproduction* journal ranked first in productivity. Keyword analysis revealed emerging hotspots: Co-culture, Androgen receptor expression, Heat stress, Testicular toxicity, and Insulin.

**Conclusion:**

This study highlights the rapid growth and global collaboration in Sertoli cell research. Current trends emphasize molecular mechanisms, environmental impacts, and therapeutic applications. Future research should prioritize interdisciplinary approaches, translational studies on male infertility, and the effects of environmental stressors on Sertoli cell function to address declining male fertility worldwide.

## 1. Introduction

Spermatogenesis constitutes a vital aspect of male reproduction, producing mature spermatozoa capable of fertilizing female ova to form zygotes. This process not only forms the basis of life continuation but also serves as a crucial indicator of male fertility. Contemporary research indicates a rising incidence of male infertility globally, with approximately 50% of cases intimately linked to spermatogenesis abnormalities.^[1,2]^ Issues such as reduced sperm count, abnormal sperm development, or compromised sperm quality (manifesting as morphological anomalies or impaired motility) rank among the primary causes of male infertility, particularly prevalent among patients with infertility lasting 5 to 10 years.^[3,4]^

Within the seminiferous tubules, Sertoli cells play a pivotal role in spermatogenesis as supportive cells. They provide structural and nutritional support to germ cells, maintain the integrity of the blood–testis barrier (BTB), and secrete proteins and hormones essential for germ cell development.^[5]^ However, Sertoli cell deficiencies can lead to spermatogenic disorders, impacting spermatogenesis and transportation processes, thereby affecting male fertility.^[6]^ Hence, understanding the functions of Sertoli cells is significant for diagnosing spermatogenesis disorders and treating male infertility.

Advancements in molecular biology have deepened researchers’ comprehension of Sertoli cell functions. Current studies primarily focus on the molecular mechanisms by which Sertoli cells sustain spermatogenesis through regulating hormones, cytokines, proteins, and signaling pathways.^[7]^ For instance, research on transcription factors and signaling pathways, particularly the regulatory role of androgens, has emerged as a focal point. Studies have revealed that androgens indirectly promote Sertoli cell maturation and functional improvement by influencing peritubular cells.^[8]^ Furthermore, environmental factors (such as endocrine disruptors and chemical pollutants) have also been shown to impair Sertoli cell function, leading to spermatogenesis abnormalities.^[9,10]^ In terms of genetic regulation, researchers are exploring key genes within Sertoli cells, where mutations or abnormal expression of genes like SRY-box transcription factor 9 and Wilms tumor 1 may contribute to reproductive disorders.^[11,12]^ Additionally, the immunoregulatory function of Sertoli cells has garnered considerable attention. Studies indicate that they not only participate in immune protection within seminiferous tubules but also modulate immune cell responses, preventing the immune system from attacking germ cells.^[13]^ Moreover, Sertoli cells can adjust their reactions to varying immune challenges, adapting to changing environments.^[5]^ Nevertheless, with advancing age, Sertoli cell function gradually declines, impacting spermatogenesis and male fertility.^[14]^

Despite the increasing number of publications on Sertoli cells and spermatogenesis, the rapid growth in literature has made it challenging for researchers to stay abreast of the latest advancements. While systematic reviews and meta-analyses provide additional informational support to researchers, they often focus on specific areas, failing to comprehensively reflect overall research trends and hotspots.^[15,16]^ Furthermore, with the rise of interdisciplinary research, studies on Sertoli cells have extended beyond reproductive biology to encompass endocrinology, toxicology, clinical medicine, and other fields, making it difficult for a single systematic review to encompass all relevant developments.^[17,18]^

Bibliometric analysis, as a research method combining quantitative and qualitative approaches, leverages tools such as VOSviewer and CiteSpace to systematically analyze extensive literature, revealing research trends, collaboration networks, and keyword evolution patterns. This, in turn, aids researchers in identifying current research hotspots and predicting future directions.^[19]^ In recent years, bibliometric analysis has been widely applied in various medical research fields, offering researchers a novel perspective and robust tool support.^[20,21]^ Through this method, researchers cannot only comprehensively grasp the research dynamics within the field but also foster interdisciplinary collaboration, driving in-depth exploration and innovative development in the area of Sertoli cells and spermatogenesis.

Given this backdrop, the present study aims to conduct a bibliometric analysis of the literature on Sertoli cells and spermatogenesis spanning from 2000 to 2024. By systematically analyzing publication trends, collaboration networks, and keyword evolution, this study seeks to map the knowledge structure of this field and identify research hotspots. This analysis will not only provide researchers with a comprehensive overview of the field but also reveal future research directions, promoting interdisciplinary collaboration and knowledge integration. Given the severe situation of declining global male fertility, a deeper understanding of the mechanistic role of Sertoli cells in spermatogenesis is particularly crucial. This study hopes to provide a scientific basis for further research in this field through bibliometric analysis and offer new insights and strategies for addressing male infertility.

## 2. Methods

### 2.1. Database selection

The Web of Science Core Collection (WoS) is a widely recognized and authoritative database in the field of medical and scientific research.^[22]^ Its robust citation analysis capabilities enable researchers to track the impact and relevance of literature, making it particularly suitable for interdisciplinary and comprehensive literature studies. To ensure the comprehensiveness and reliability of the retrieval results, we have selected WoS as the primary data source for this study.

### 2.2. Literature search strategy and time frame

**Figure 1.**
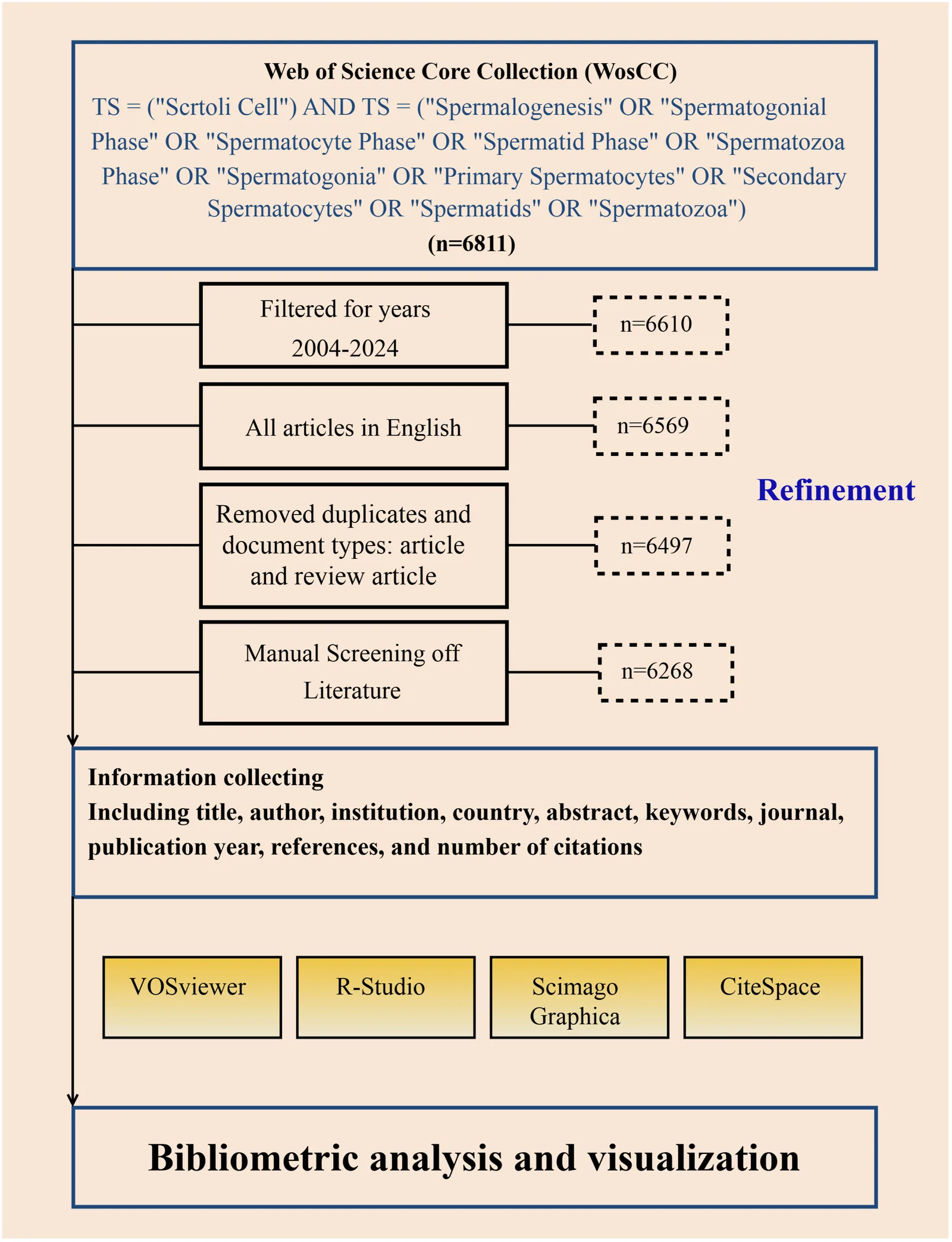
Data extraction process.

Establishing precise literature screening criteria is crucial for ensuring the quality and relevance of the review. By applying rigorous selection criteria, researchers can focus on the most relevant and influential literature, thereby laying a solid foundation for subsequent analysis and discussion. In the literature search for this study, we employed the following search terms: Topic = (“Sertoli Cell”) AND Topic = (“Spermatogenesis” OR “Spermatogonial Phase” OR “Spermatocyte Phase” OR “Spermatid Phase” OR “Spermatozoa Phase” OR “Spermatogonia” OR “Primary Spermatocytes” OR “Secondary Spermatocytes” OR “Spermatids” OR ‘Spermatozoa”). The search time frame was set from 2000 to 2024. Only original research articles and reviews with standard references and peer-reviewed were included. Ultimately, 6811 records were retrieved and exported in “plain text” format, containing “complete records and references.” The processing workflow is illustrated in Figure 1.

### 2.3. Statistical analysis

For the bibliometric analysis in this study, we utilized Microsoft Excel 2016 and 4 scientific metrology software tools, including R-Studio (Version 4.4.1, http://www.bibliometrix.org), VOSviewer (Version 1.6.16, https://www.vosviewer.com/download), CiteSpace (Version 6.3R1, https://citespace.podia.com/courses/download), and Scimago Graphica (Version 1.0.42, https://graphica.app/download).^[23]^ During the data processing phase, synonyms and semantically similar terms were consolidated to enhance the accuracy of the analysis. This consolidation followed the Medical Subject Headings vocabulary standards, with 1 researcher (Anan Yu) independently reviewing and cross-checking all keywords; any disagreements were resolved by the corresponding author (Hongyu Chen), who made the final determination.

## 3. Results

### 3.1. Number of studies and publication trends

**Figure 2.**
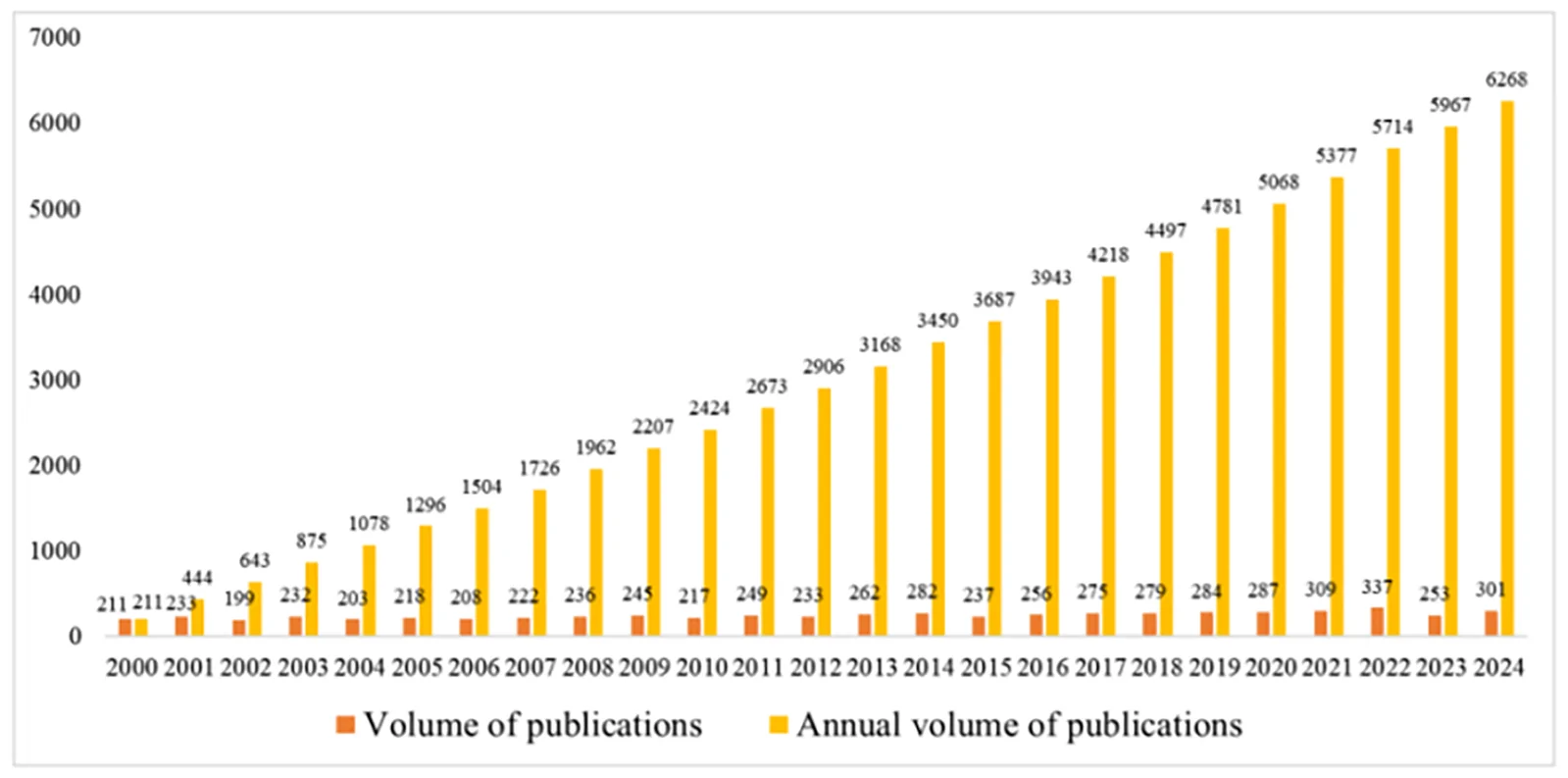
The number of annually published articles and cumulative number of published articles.

This study retrieved a total of 6811 articles from the WoS database. After data processing, 6268 relevant articles, including original research papers and reviews, were ultimately included. These articles span from 2000 to 2024, with Figure 2 providing a visualization of the annual and cumulative publication volumes. The results indicate that in 2000, there were 211 publications in the field of Sertoli cells and spermatogenesis, while by 2024, the cumulative publication volume had reached 6268 articles, demonstrating the continuous accumulation of research outcomes in this field and providing a rich literature foundation for subsequent studies.

Specifically, from 2000 to 2006, this field was in its early stages, with a relatively low and slowly increasing number of publications. Beginning in 2007, the publication volume showed a gradual upward trend, suggesting that the field was gradually gaining the attention of more researchers. After reaching a peak in 2022, the publication volume fluctuated slightly in 2023 but rapidly resumed growth in 2024, indicating that the research heat in this field remains undiminished.

### 3.2. Country analysis

**Figure 3.**
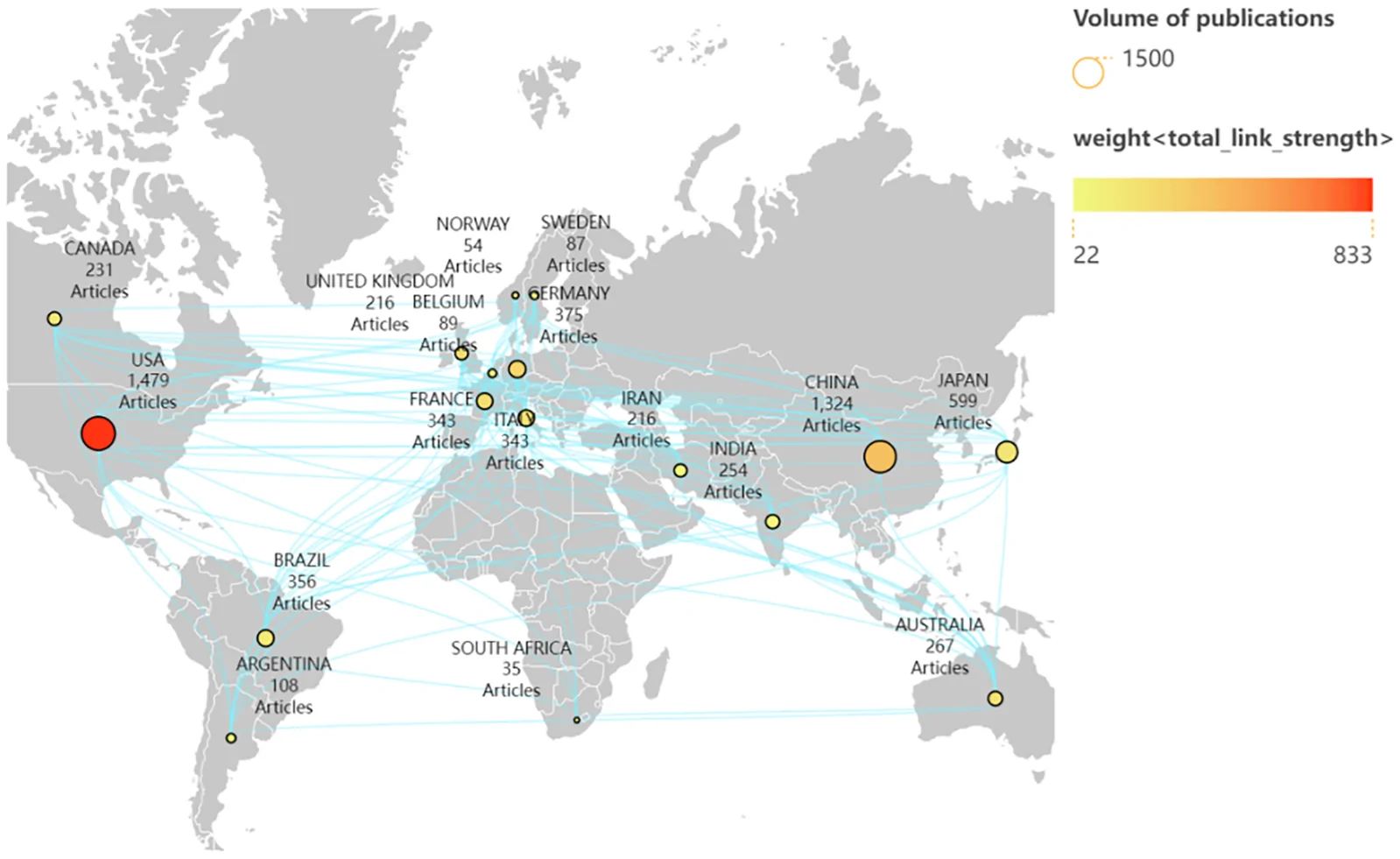
National visualization network map.

In the field of Sertoli cells and spermatogenesis, a total of 93 countries have contributed to the research. By analyzing the publication volume, we found that the United States and China lead the field with 1479 and 1324 publications, respectively. Other countries, such as Japan (n = 599), Germany (n = 375), Brazil (n = 356), France (n = 343), Italy (n = 343), Australia (n = 267), India (n = 254), and Canada (n = 231), have also made significant contributions (Table 1). To visually present the collaboration among countries, we used VOSviewer and Scimago Graphica to create a country collaboration network diagram (Fig. 3). The color and thickness of the lines in the diagram represent the intensity of collaboration between countries, with the United States occupying a central position in the global research collaboration network, demonstrating its leadership role in this field.

**Table 1:** Top 10 countries with the highest productivity.

Rank	Countries	Publications	Centrality	Area
1	USA	1479	0.28	North America
2	China	1324	0.09	Asia
3	Japan	599	0.14	Asia
4	Germany	375	0.18	Europe
5	Brazil	356	0.05	South America
6	France	343	0.15	Europe
7	Italy	343	0.19	Europe
8	Australia	267	0.06	Oceania
9	India	254	0.02	Asia
10	Canada	231	0.06	North America

### 3.3. Institutional analysis

**Figure 4.**
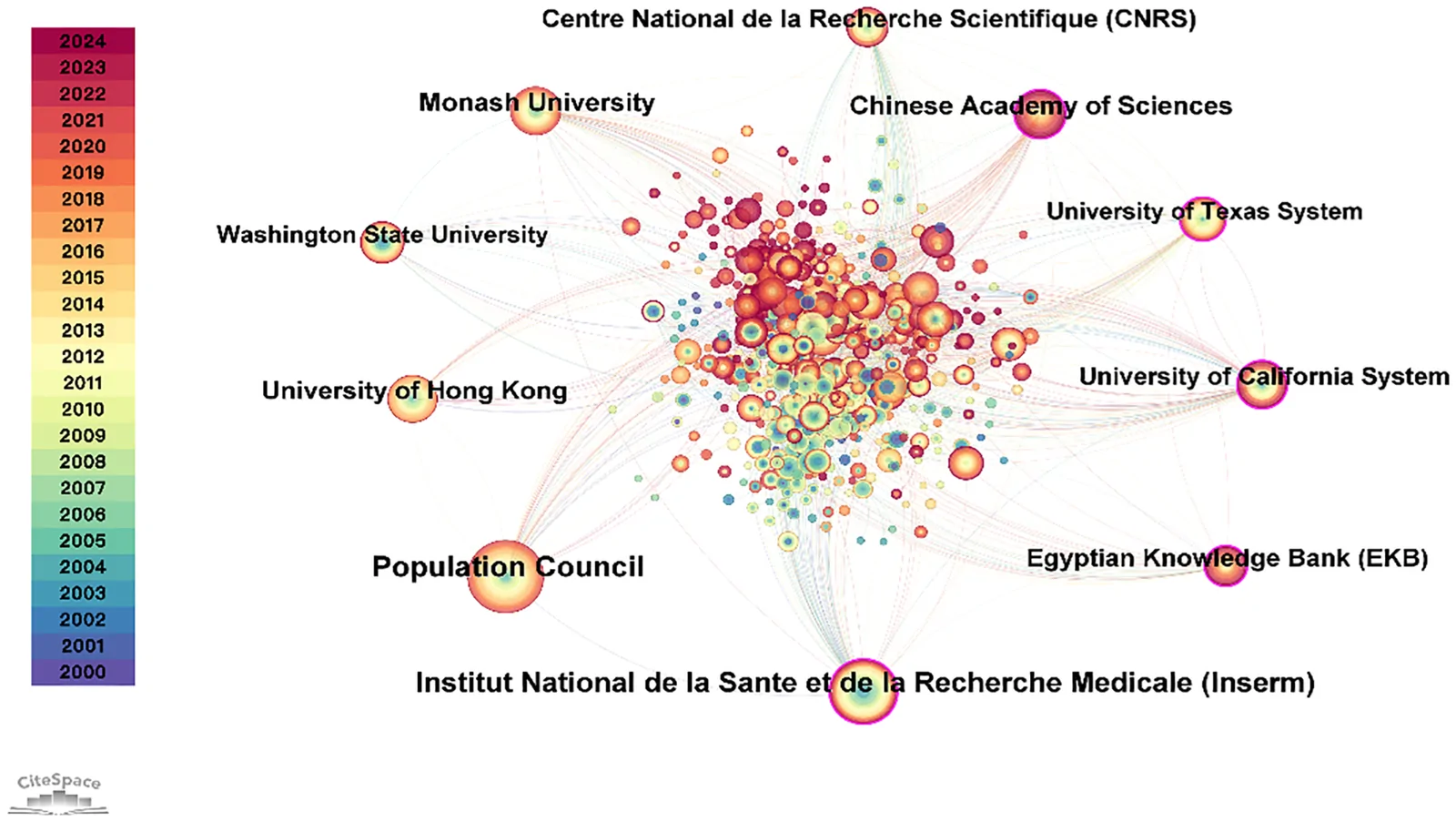
The main collaboration network of institutions.

The productivity and contribution of research institutions are important indicators of their research quality. Through literature analysis, we found that 469 research institutions have contributed to the field of Sertoli cells and spermatogenesis. Table 2 lists the top 10 institutions with the highest publication volumes, primarily located in the United States, France, and China. Among them, the Population Council in the USA and Inserm (Institut National de la Sante et de la Recherche Medicale) in France rank at the top with 263 and 206 publications, respectively. Interestingly, although the Population Council has the highest publication volume, its centrality is relatively low at 0.09, indicating limited collaboration and influence within the network. In contrast, Inserm (Centrality = 0.19), the Chinese Academy of Sciences (Centrality = 0.19), and the University of California System (Centrality = 0.18) occupy core positions in the collaboration network and have significant influence. The institutional collaboration network diagram created by Citespace (Fig. 4) further demonstrates the collaboration relationships among these institutions and their connectivity within the global research network.

**Table 2:** The top 10 institutions with high publications.

Rank	Institutions	Publications	Centrality	Country
1	Population Council	263	0.09	USA
2	Institut National de la Sante et de la Recherche Medicale (Inserm)	206	0.19	France
3	Monash University	138	0.09	Australia
4	Chinese Academy of Sciences	132	0.19	China
5	University of Hong Kong	116	0.01	China
6	Centre National de la Recherche Scientifique (CNRS)	114	0.09	France
7	Egyptian Knowledge Bank (EKB)	104	0.12	Egypt
8	University of California System	100	0.18	USA
9	University of Texas System	88	0.11	USA
10	Washington State University	87	0.02	USA

### 3.4. Author analysis

**Figure 5.**
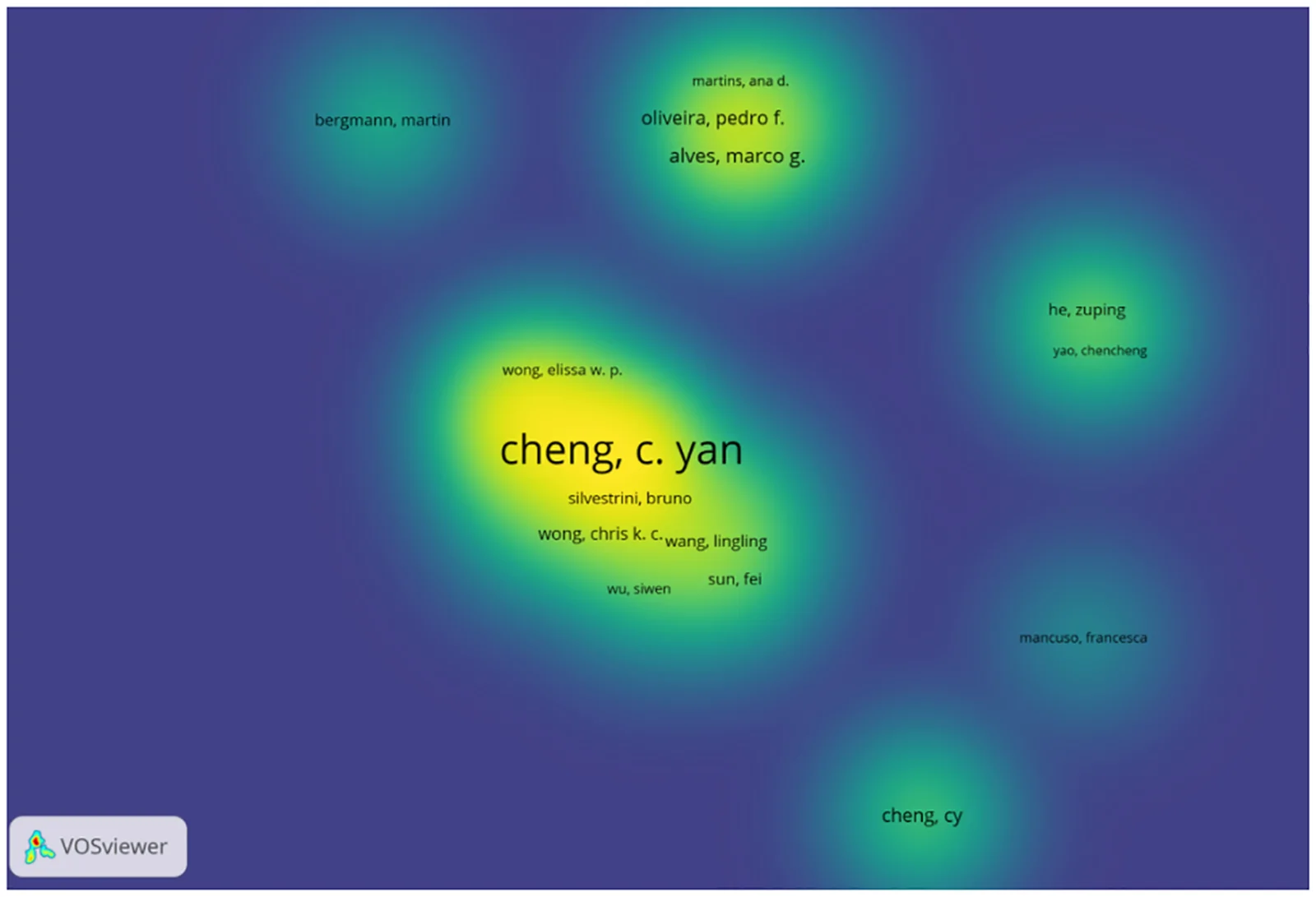
Density visualization of core authors.

A total of 1143 authors have participated in the research on Sertoli cells and spermatogenesis. According to Lotka Law, a small group of core authors often contributes the highest productivity.^[24]^ Price’s criteria for becoming a core author is set at n = 0.749 √ Nmax, where Nmax is the publication count of the most productive author.^[25]^ Table 3 lists the top 10 authors with the highest publication volumes, with Cheng, C. Yan leading with 197 publications, followed by Mruk, Dolores D. with 115. Their research focuses on the complex interactions between various types of cell junctions in Sertoli cells and germ cells. These studies are crucial for understanding how germ cells are correctly positioned and move within the testes and have significant implications for the development of novel male contraceptives.^[26,27]^ The author density visualization diagram is shown in Figure 5, which demonstrates that through resource integration, team optimization, and enhanced international collaboration, core authors and their research groups have significantly increased their research output and influence.

**Table 3:** The top 10 most productive authors.

Rank	Authors	Papers	Year
1	Cheng, C Yan	197	2006
2	Mruk, Dolores D	115	2006
3	Lee, Will M	65	2006
4	Alves, Marco G	52	2012
5	Cheng, CY	49	2000
6	Oliveira, Pedro F	48	2013
7	Wong, Chris K C	36	2013
8	Sun, Fei	29	2020
9	Li, Linxi	28	2017
10	Bergmann, Martin	25	2006

### 3.5. Journal analysis

**Figure 6.**
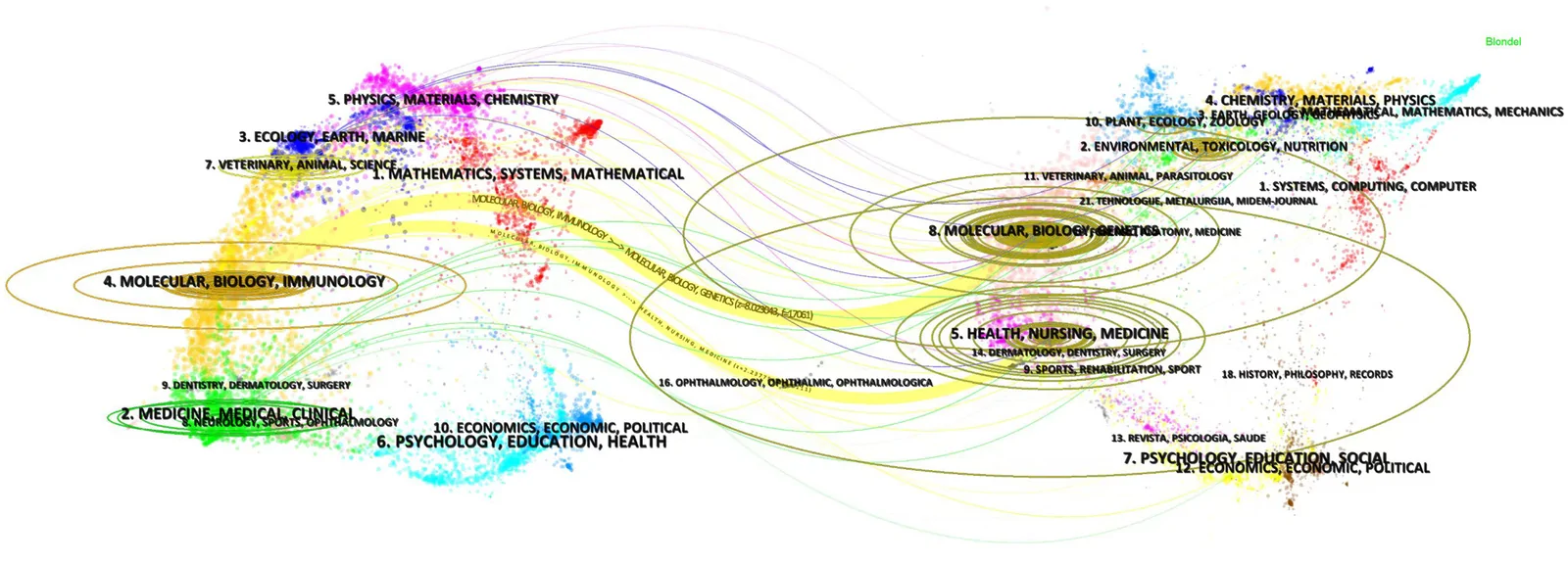
Dual-map overlays.

Scientific publications play a key role in transmitting discipline-specific knowledge. Highly cited papers, often featuring innovative research methods or significant clinical discoveries, tend to attract widespread attention and discussion. Through statistical analysis of journal sources, we identified the top 10 journals with the highest publication volumes in this field (Table 4). Among them, *Biology of Reproduction*, with 4080 citations, ranks first and is one of the most influential journals in this field. To further demonstrate the citation relationships among journals, we created a dual-map overlays diagram (Fig. 6). Figure 6 shows that in the field of Sertoli cell-related spermatogenesis, citing journals mainly come from 2 different disciplines, while cited journals are concentrated in 1 discipline. The core citation path (Table 5) reveals that journals in the field of *Molecular Biology and Immunology* frequently cite journals in the field of *Molecular Biology and Genetics*, totaling 31,602 citations. This citation pattern has a *Z*-score of 8.598917, indicating that this citation relationship is statistically significant and reflecting the close academic ties and mutual influence between these 2 fields.

**Table 4:** The top 10 most productive journals.

Rank	Full journal title	Citations	IF2024	WOS categories
1	*Biology of Reproduction*	4080	3.1	Reproductive Biology
2	*Endocrinology*	3742	3.8	Endocrinology & Metabolism
3	*Proceedings of the National Academy of Sciences of the United States of America*	3510	9.4	Multidisciplinary Sciences
4	*Journal of Andrology*	2874	0	Afterwards
5	*Journal of Biological Chemistry*	2639	4	Biochemistry & Molecular biology
6	*Reproduction*	2522	3.7	Developmental Biology
7	*Nature*	2438	50.5	Multidisciplinary Sciences
8	*Molecular and Cellular Endocrinology*	2245	3.8	Cell Biology
9	*Science*	2153	44.8	Multidisciplinary Sciences
10	*Human Reproduction*	2150	6	Obstetrics & Gynecology

**Table 5:** Main citation path statistics of dual-map overlays.

Rank	Citing journal cluster	Cited journal cluster	Citations	*Z* score
1	Molecular, Biology, Immunology	Molecular, Biology, Genetics	31,602	8.598917
2	Molecular, Biology, Immunology	Health, Nursing, Medicine	9259	2.359443

### 3.6. Analysis of keywords

**Figure 7.**
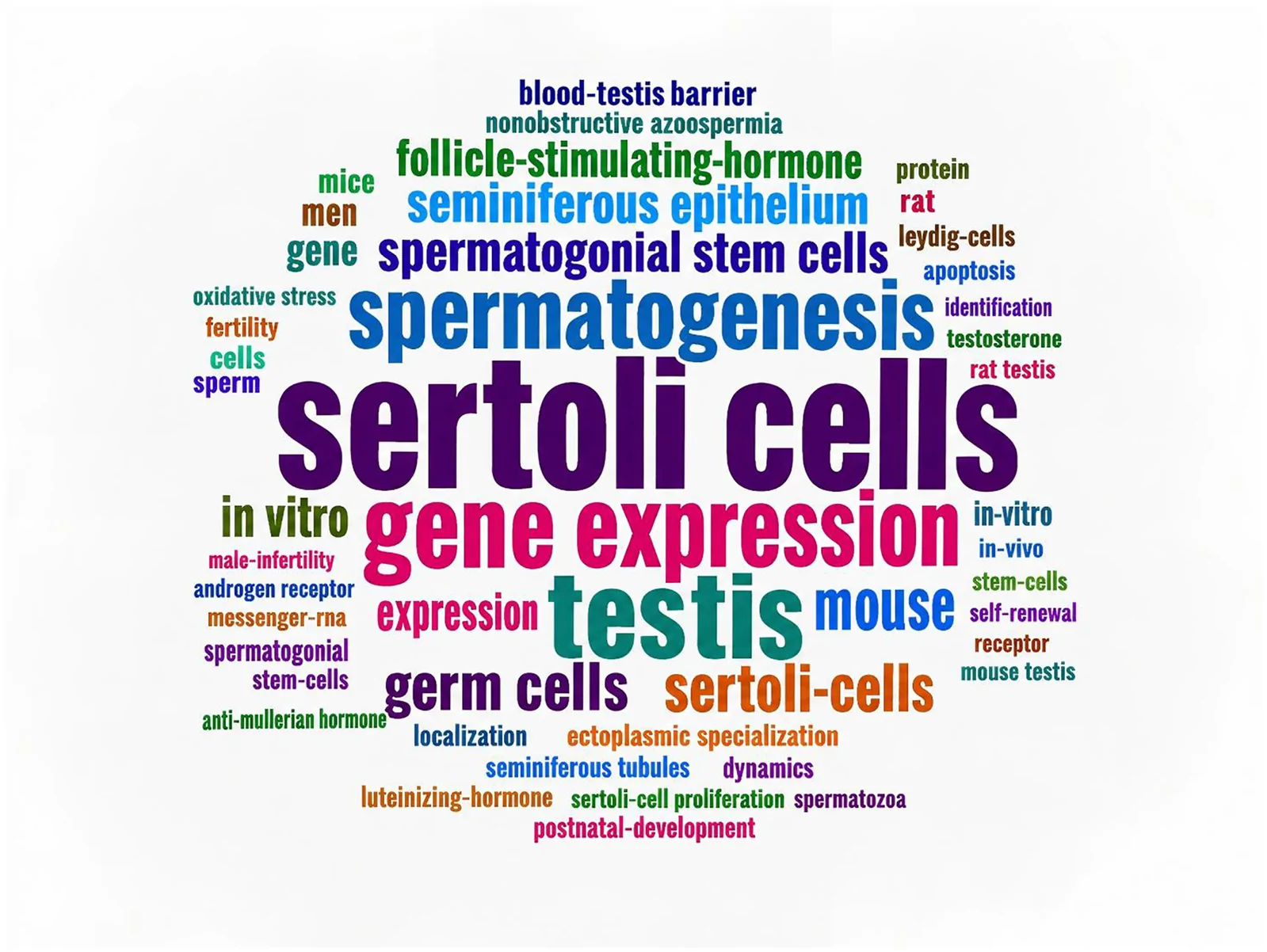
Word cloud.

This analysis extracted a total of 939 keywords, showcasing the research hotspots in the field of Sertoli cells and spermatogenesis. Table 6 lists the top 10 most frequent keywords, with “Sertoli Cells,” “Gene Expression,” and “Spermatogenesis” ranking at the top. A word cloud further visually represents the distribution of these keywords (Fig. 7).

**Table 6:** The top 10 keywords with high frequency.

Rank	Keywords	Frequency
1	Sertoli cells	3039
2	Gene expression	1949
3	Spermatogenesis	1603
4	Testis	1575
5	Germ cells	790
6	Mouse	713
7	Follicle stimulating hormone	556
8	Seminiferous epithelium	551
9	Spermatogonial stem cells	522
10	In vitro	497

**Figure 8.**
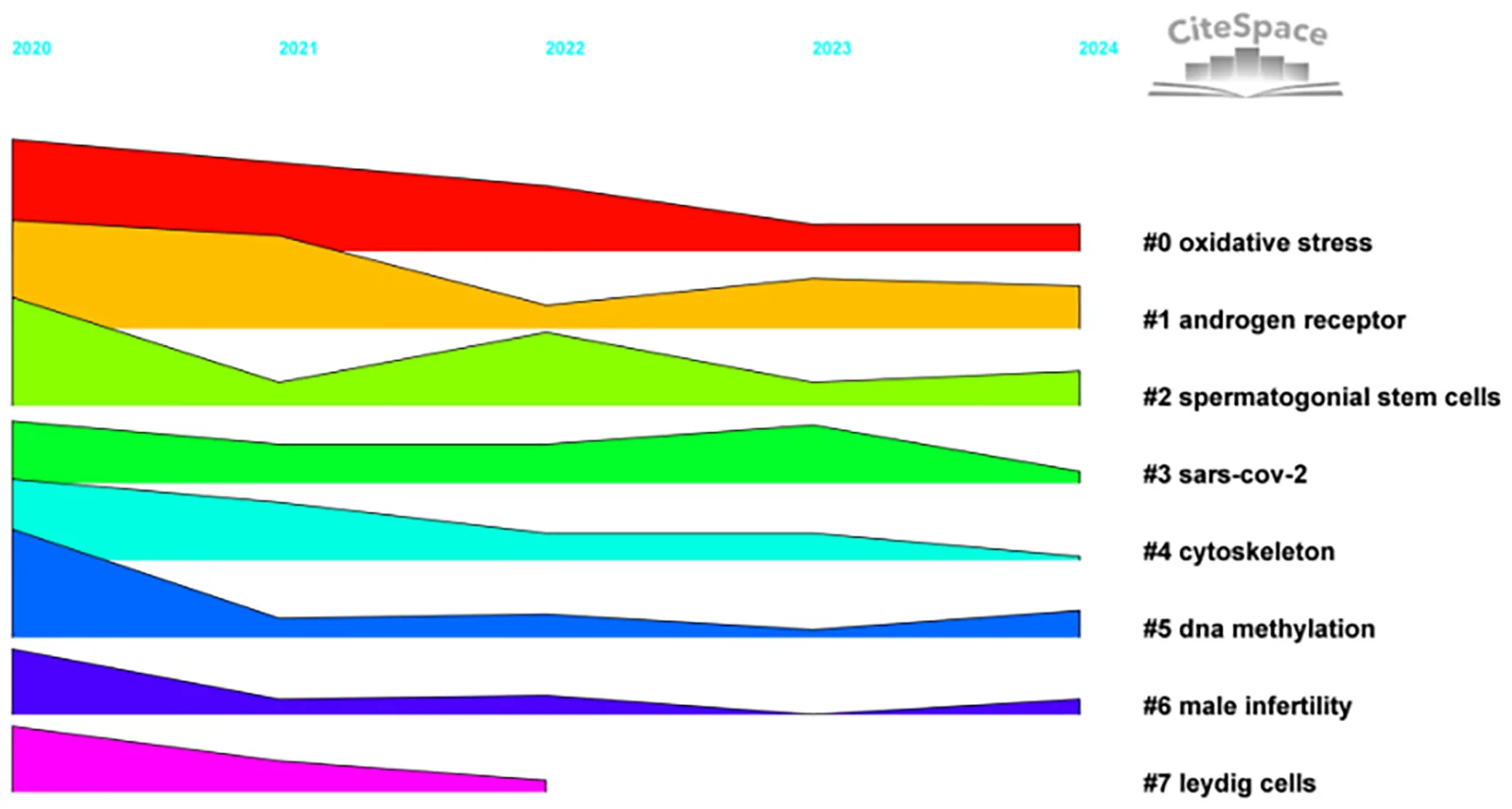
Landscape.

**Figure 9.**
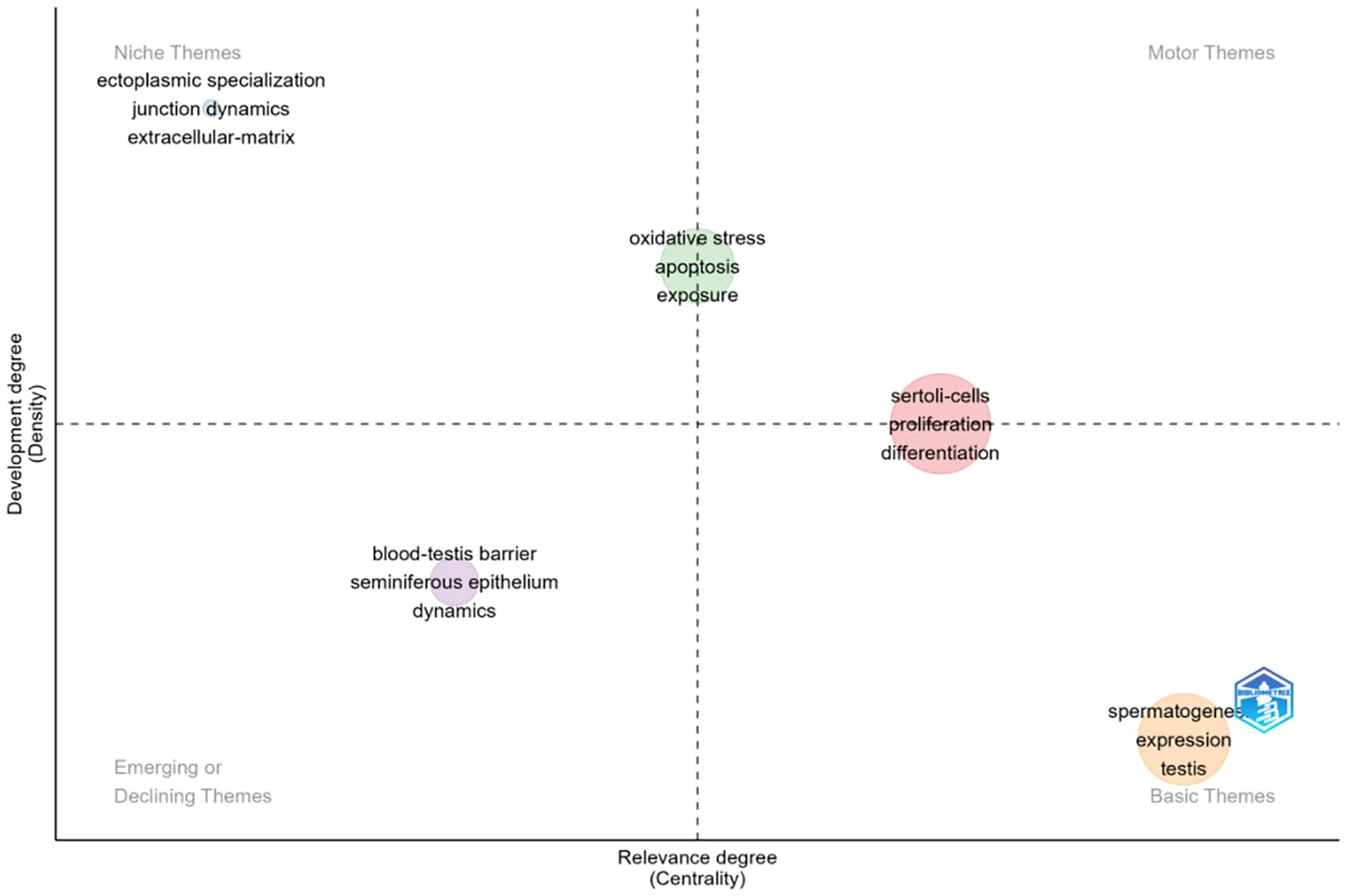
Thematic map.

To gain insights into recent research hotspots, we created a keyword clustering diagram for the years 2020 to 2024 (Fig. 8). The analysis shows that #0 Oxidative Stress, #1 Androgen Receptor, #2 Spermatogonial Stem Cells, #3 SARS-CoV-2, #4 Cytoskeleton, #5 DNA Methylation, and #6 Male Infertility constitute the main research clusters. Additionally, using the bibliometrix program, we created a thematic map of keywords for the years 2020 to 2024 (Fig. 9). The results indicate that “spermatogenesis,” as the foundational theme, is connected to multiple other themes and serves as the cornerstone of research. Themes such as “cytoplasmic specialization,” “junction dynamics,” and “extracellular matrix” may be important within specific research areas but have a narrower coverage across the entire dataset. Themes like “blood-testis barrier,” “seminiferous epithelium,” and “dynamics” located in the lower left corner may represent emerging research directions or areas gradually losing attention. “Sertoli cells,” “proliferation,” and “differentiation” are located on the right side of the map, showing their high relevance in the research field and their close connections with multiple other themes.

**Figure 10.**
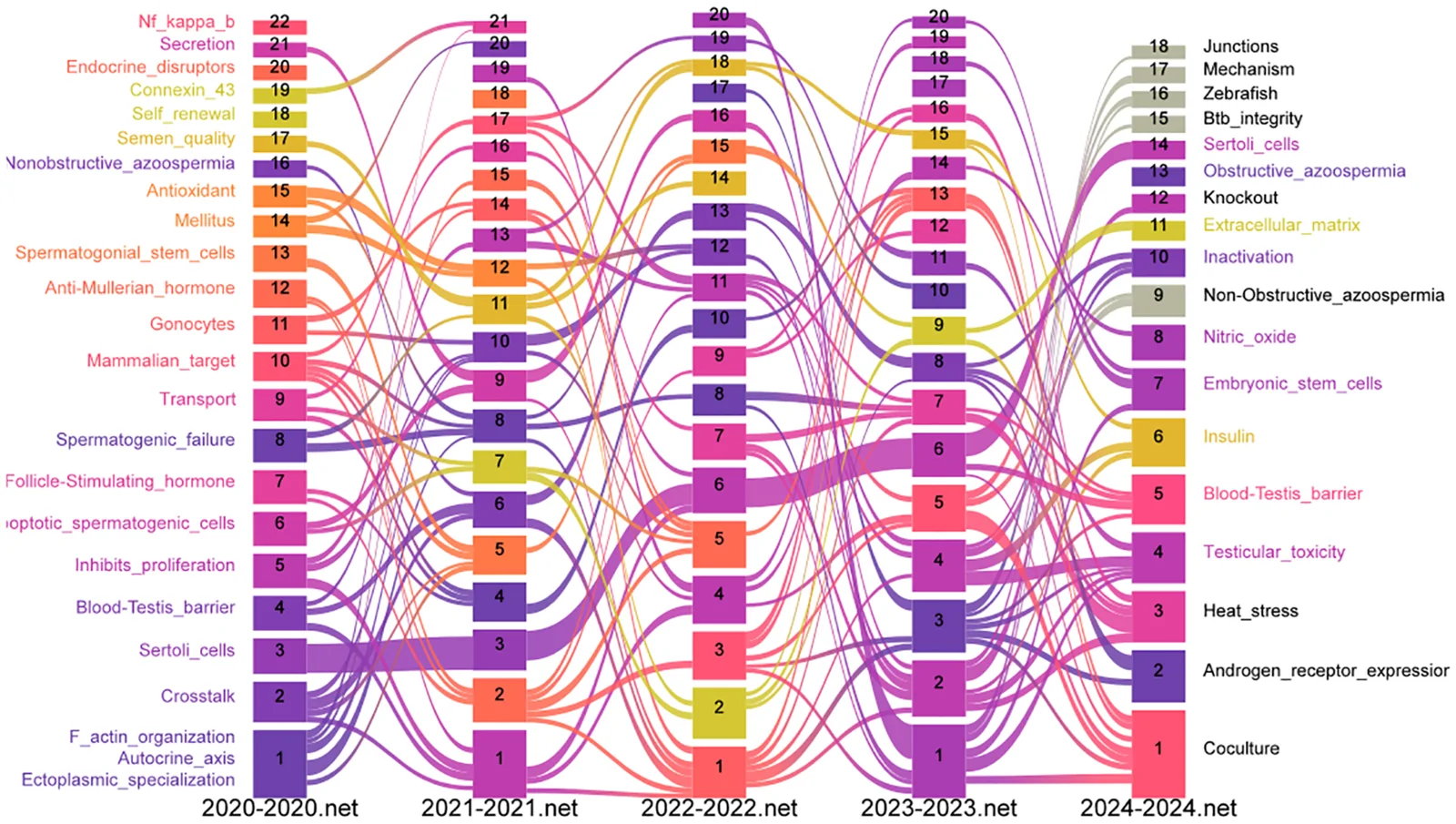
Sankey diagram.

Subsequently, we used a Sankey diagram (Fig. 10) to demonstrate the evolution and associations of research themes from 2020 to 2024, revealing that “Sertoli cells” and the “Blood-Testis Barrier” they form remain core research themes in the field of spermatogenesis. Meanwhile, emerging hotspots such as “Co-culture,” “Androgen receptor expression,” “Heat stress,” “Testicular toxicity,” and “Insulin” are gradually gaining attention.

## 4. Discussion

In recent years, remarkable advancements have been made in the study of Sertoli cells in spermatogenesis, particularly in the context of male infertility.^[18,28]^ The growing body of literature on Sertoli cells reflects sustained research interest in this area, likely motivated in part by the clinical significance of male infertility, given the pivotal roles of Sertoli cells in supporting germ cell development and maintaining the BTB.^[29,30]^ This research conducted a bibliometric analysis of studies on Sertoli cells and spermatogenesis from 2000 to 2024, comprehensively revealing research trends, collaboration networks, and emerging hotspots in this field.

Our findings indicate a steady output of publications in the field of Sertoli cells and spermatogenesis, reflecting the growing recognition of the central role of Sertoli cells in male reproductive health. The peak publication volume in 2022 underscores the intense research activity during this period. This growth is likely driven by the rising incidence of male infertility, prompting researchers to explore the underlying mechanisms of spermatogenesis dysfunction. Data from China’s sperm banks in 2023 revealed that only 19% of sperm donors met quality standards, highlighting the urgency of addressing male reproductive health issues.^[31]^ Meanwhile, projections by institutions such as the University of Washington suggest that with the continued decline in fertility rates, global population growth will peak in 2064 and subsequently decline, with significant implications for the labor market and economic stability, particularly in Europe and Asia.^[32]^ These demographic trends, coupled with environmental stressors like COVID-19, further emphasize the importance of studying Sertoli cell function and its role in male fertility.^[33]^

Moreover, our results show that Sertoli cell research has formed multilayered collaboration networks, reflecting close ties among countries, institutions, and researchers. The United States and China lead in research output. Institutions such as the Population Council, Inserm, and the Chinese Academy of Sciences have made significant contributions to advancing research on Sertoli cell–germ cell interactions and developing novel therapeutic strategies. Additionally, Sertoli cell research exhibits a notable interdisciplinary nature, spanning reproductive biology, endocrinology, toxicology, and clinical medicine, among others. This integration links Sertoli cells with areas such as male infertility, environmental toxin effects, and hormonal regulation, thereby facilitating the convergence of diverse perspectives and methodologies.^[9,34–36]^ For example, a landmark study demonstrated that glial cell line-derived neurotrophic factor, produced by Sertoli cells, regulates the fate determination of spermatogonial stem cells in a dose-dependent manner: appropriate levels maintain the stem cell reserve, whereas overexpression leads to accumulation of undifferentiated spermatogonia and testicular tumor formation.^[37]^ Similarly, recent studies have explored the impact of environmental toxins on Sertoli cell function, revealing how pollutants like copper (Cu) and polystyrene nanoparticles disrupt the BTB and impair spermatogenesis.^[38,39]^ These findings underscore the importance of interdisciplinary collaboration in addressing complex reproductive health challenges.

**Figure 11.**
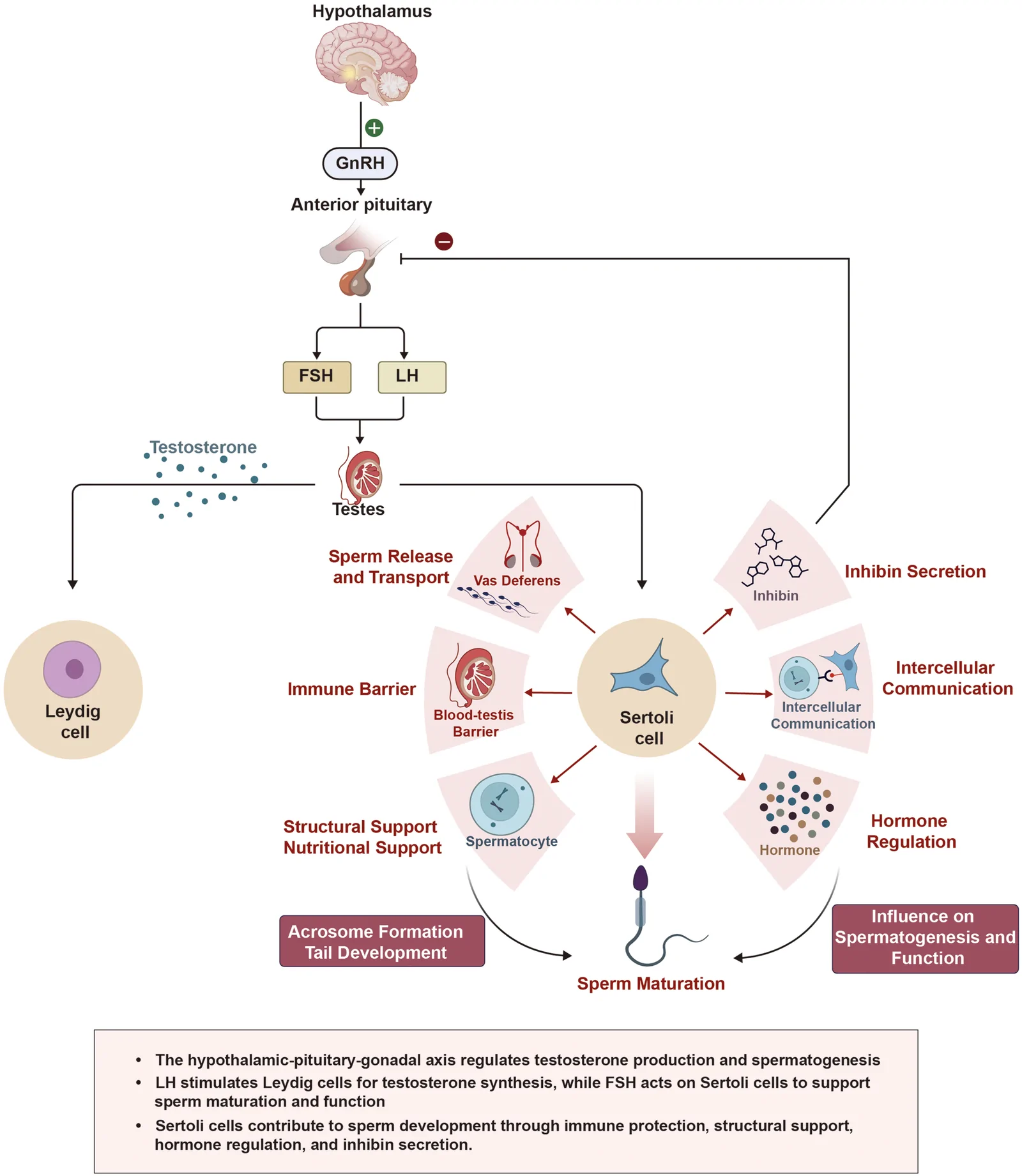
Diagram of the spermatogenic mechanism of Sertoli cells. FSH = follicle-stimulating hormone, LH = luteinizing hormone.

By examining the evolution of research hotspots and keywords, we further elucidated current research focuses and future trends in Sertoli cell studies. In recent years, a major hotspot in Sertoli cell research during spermatogenesis has centered on “proliferation and differentiation of Sertoli cells.” The proliferation and differentiation of Sertoli cells are regulated by various factors. Studies have found that the hypothalamic–pituitary–gonadal axis stimulates the anterior pituitary to release follicle-stimulating hormone and luteinizing hormone by secreting gonadotropin-releasing hormone from the hypothalamus. In fetuses and early postnatal life, follicle-stimulating hormone directly regulates the proliferation and differentiation of Sertoli cells, ensuring an adequate number of cells to support spermatogenesis during early reproductive development.^[40]^ Luteinizing hormone also indirectly affects Sertoli cell proliferation and differentiation by stimulating Leydig cells to secrete testosterone, enabling Sertoli cells to provide immune protection, structural support, hormonal regulation, and secretion of inhibin, and forming the BTB to protect developing spermatozoa from immune system attacks, as illustrated in Figure 11.^[7]^ Additionally, multiple signaling pathways influence Sertoli cells during spermatogenesis, such as the mitogen-activated protein kinase pathway, which controls Sertoli cell proliferation and survival by regulating the expression of cyclins and cyclin-dependent kinase inhibitors, and the phosphoinositide 3-kinase/protein kinase B (PI3K/Akt) pathway, which promotes the proliferation and anti-apoptosis of immature Sertoli cells and maintains and promotes spermatogenesis in male testes directly or indirectly by regulating protein synthesis and disrupting the BTB structure through cytoskeletal rearrangement in Sertoli cells.^[41,42]^

Furthermore, keywords such as “Co-culture,” “Androgen receptor expression,” “Heat stress,” “Testicular toxicity,” and “Insulin” are currently attracting attention from researchers in the field and may show an upward trend in future studies. Co-culture techniques, involving the simultaneous culturing of Sertoli cells with other cell types (e.g., mesenchymal stem cells or embryonic stem cells), have shown potential in creating testicular organoids that mimic the in vivo environment.^[43,44]^ These models provide valuable insights into the cellular interactions regulating spermatogenesis and serve as potential platforms for drug screening and toxicity testing. The role of the androgen receptor in Sertoli cell function has garnered significant attention, with studies indicating its crucial role in regulating cell proliferation, differentiation, and BTB integrity.^[45,46]^ Understanding the molecular mechanisms of androgen receptor signaling may pave the way for novel therapeutic interventions for male infertility. Heat stress and testicular toxicity are also key areas for future research, especially in the context of environmental and occupational exposures. Heat stress has been shown to disrupt the BTB, increase oxidative stress, and induce Sertoli cell apoptosis, ultimately impairing spermatogenesis.^[47]^ Similarly, environmental toxins such as di(2-ethylhexyl) phthalate and polystyrene nanoparticles are associated with testicular damage and decreased sperm quality, emphasizing the need to mitigate these effects.^[39,48]^ These findings highlight the potential to improve male reproductive health by targeting metabolic pathways. In recent years, emerging areas of research have included the pathogenesis of SARS-CoV-2 in the testis: Sertoli cells express the viral entry receptors ACE2 and TMPRSS2,^[49]^ and autopsy studies have confirmed seminiferous tubular damage, Sertoli-cell-only-like histology, and detectable viral particles in testicular tissue.^[50,51]^ Meta-analyses have now established significant postinfection reductions in sperm concentration, motility, and testosterone levels, substantiating male reproductive sequelae as a clinically important consequence of COVID-19.^[52,53]^ Concurrently, insulin signaling has been identified as a metabolic rheostat of Sertoli cell function. For instance, insulin regulates lactate production through the IRS2/PI3K/Akt and GLUT3/LDHA/MCT1 axis, a pathway indispensable for germ cell energy metabolism^[54]^; Sertoli cells express the insulin-responsive glucose transporters GLUT1, GLUT3, and GLUT4, whose membrane translocation depends on PI3K/Akt signaling.^[55]^ Insulin and insulin-like growth factors have also emerged as key regulators of Sertoli cell function, with evidence suggesting they promote cell survival and support spermatogenesis through the PI3K/Akt signaling pathway.^[40,42]^

Based on these insights, researchers are currently advancing innovative therapies at the cellular, tissue, and systemic levels, with a focus on supporting cell regulation and novel treatment strategies. These advancements not only deepen our understanding of the role of Sertoli cells in spermatogenesis but also refine clinical approaches to addressing male infertility. As shown in Table 7, key progress in targeted therapy for Sertoli cells is systematically summarized, elucidating the mechanistic levels and priority translation strategies. Based on the aforementioned research progress, the author proposes the following suggestions aimed at promoting clinical translational research on male infertility through innovative therapeutic strategies and interdisciplinary collaboration, offering new hope for improving global male reproductive health: develop new therapies to restore or enhance the function of the BTB: By repairing or strengthening the integrity of the BTB, improve the microenvironment for spermatogenesis, thereby enhancing sperm quality and quantity.^[5]^ Utilize hormone regulation to promote Sertoli cell function for the treatment of hormone-related infertility.^[7]^ Develop therapies based on the immunomodulatory capacity of Sertoli cells: leverage the immune-privileged properties of Sertoli cells to design novel immunomodulatory therapies for the treatment of immune-related infertility, preventing the immune system from attacking germ cells.^[56]^ Develop drugs to protect Sertoli cells: in response to the damage to Sertoli cells caused by environmental toxins (such as heavy metals and nanoparticles), develop protective drugs or therapies to reduce the negative impact of environmental factors on spermatogenesis.^[39]^ Utilize co-culture technology to mimic the testicular microenvironment for drug screening and toxicity testing, and develop new treatment methods for infertility.^[57]^ Drug development targeting the androgen receptor signaling pathway: deeply investigate the role of the androgen receptor in Sertoli cell function and develop drugs that target this signaling pathway to improve the treatment outcomes of male infertility.^[58]^ Develop antioxidants and cooling therapies: Addressing the damage to Sertoli cells caused by heat stress and oxidative stress, develop antioxidants or cooling therapies to protect Sertoli cell function and improve the process of spermatogenesis.^[59]^ Modulate the insulin signaling pathway to improve Sertoli cell function: By regulating the insulin and insulin-like growth factor signaling pathways, promote the survival and metabolic function of Sertoli cells for the treatment of metabolism-related infertility.^[60]^ Develop antiaging therapies to delay the aging of Sertoli cells: in response to the functional decline of Sertoli cells with age, develop antiaging therapies to delay cellular aging and enhance the fertility of elderly men.^[29]^ By promoting interdisciplinary collaboration and leveraging emerging technologies, the scientific community can make significant progress in addressing the challenges of male infertility and improving global reproductive health, ultimately translating basic research into effective clinical interventions and bringing new hope to male reproductive health.

**Table 7:** Key advances and therapeutic applications in sertoli cell-targeted therapy.

Research area	Key mechanisms	Potential therapeutic applications	References
Blood–testis barrier function	Sertoli cells form the blood–testis barrier, protecting germ cells from immune attacks and toxins.	Restore or enhance blood–testis barrier function to improve the spermatogenic environment.	DOI: 10.3389/fimmu.2022.913502
Hormonal regulation	Sertoli cells respond to FSH and testosterone, regulating spermatogenesis.	Promote Sertoli cell function through hormonal regulation to treat hormone-related infertility.	DOI: 10.3389/fendo.2021.648141
Immune regulation	Sertoli cells have immune privilege, preventing immune system attacks on germ cells.	Utilize Sertoli cell immune regulation to treat immune-related infertility.	DOI: 10.1016/j.semcdb.2021.06.016
Environmental toxins	Environmental toxins (e.g., heavy metals, nanoparticles) impair Sertoli cell function, leading to infertility.	Develop drugs or therapies to protect Sertoli cells and reduce the impact of environmental toxins on spermatogenesis.	DOI: 10.1016/j.envpol.2021.117904
Co-culture techniques	Co-culture of Sertoli cells with other cells (e.g., mesenchymal stem cells) mimics the testicular microenvironment.	Used for drug screening and toxicity testing, developing novel infertility treatments.	DOI: 10.1007/s00204-024-03767-6
Androgen receptor Signaling	Androgen receptors play a key role in Sertoli cell proliferation, differentiation, and blood–testis barrier integrity.	Target androgen receptor signaling pathways to develop drugs for male infertility.	DOI: 10.3389/fendo.2022.838858
Heat and oxidative stress	Heat and oxidative stress impair Sertoli cell function, leading to spermatogenic disorders.	Develop antioxidants or cooling therapies to protect Sertoli cells from stress damage.	DOI: 10.1016/j.theriogenology.2023.10.018
Insulin signaling pathway	Insulin and IGF promote Sertoli cell survival and spermatogenesis through the PI3K/Akt pathway.	Modulate insulin signaling pathways to improve Sertoli cell function and treat metabolism-related infertility.	DOI: 10.3389/fendo.2022.1010796
Aging and apoptosis	With aging, Sertoli cell function declines, leading to reduced spermatogenesis.	Develop antiaging therapies to delay Sertoli cell decline and improve fertility in aging males.	DOI: 10.3389/fendo.2022.1012119

FSH = follicle-stimulating hormone, IGF = insulin-like growth factor, PI3K/Akt = phosphoinositide 3-kinase/protein kinase B.

Despite providing a comprehensive analysis of the field of Sertoli cells and spermatogenesis, this study has some limitations. Firstly, it primarily relies on the WOS database, potentially missing relevant publications from other databases. Future research should consider including additional databases such as PubMed and Scopus to enhance the comprehensiveness of literature retrieval. Secondly, this study focuses solely on English-language publications, potentially overlooking important research from non-English-speaking countries. Future studies should broaden the language scope to more comprehensively reflect global research trends. Additionally, while bibliometric analysis can reveal research hotspots and trends, it cannot delve deeply into specific research content. Therefore, future research should combine qualitative analysis methods, such as systematic reviews and meta-analyses, to gain a deeper understanding of the specific mechanisms of Sertoli cells in spermatogenesis.

In conclusion, this bibliometric analysis provides valuable insights into the research trends and hotspots within the fields of Sertoli cell biology and spermatogenesis. The findings underscore the critical role of international collaboration, interdisciplinary strategies, and the integration of cutting-edge technologies in advancing male reproductive health research. Given the escalating global burden of male infertility, future research may benefit from an increased emphasis on the development of innovative diagnostic and therapeutic approaches, with particular attention to improving patient outcomes and addressing the root causes of reproductive dysfunction. Through the promotion of interdisciplinary cooperation and the application of emerging technologies, the scientific community may discover new research value in tackling the challenges of male infertility and enhancing reproductive health worldwide.

## Acknowledgments

We would like to thank the anonymous reviewers for their helpful remarks.

## Author contributions

**Formal analysis:** Anan Yu.

**Funding acquisition:** Hongyu Chen.

**Investigation:** Anan Yu.

**Methodology:** Anan Yu.

**Software:** Hongyu Chen.

**Supervision:** Hongyu Chen.

**Validation:** Hongyu Chen.

**Visualization:** Anan Yu.

**Writing – original draft:** Anan Yu.

**Writing – review & editing:** Hongyu Chen.

## References

[1] Liang Y Huang J Zhao Q. Global, regional, and national prevalence and trends of infertility among individuals of reproductive age (15–49 years) from 1990 to 2021, with projections to 2040. Hum Reprod. 2025;40:529–44. 39752330 10.1093/humrep/deae292

[2] Foran D Chen R Jayasena CN Minhas S Tharakan T. The use of hormone stimulation in male infertility. Curr Opin Pharmacol. 2023;68:102333. 36580771 10.1016/j.coph.2022.102333

[3] Chao HH Zhang Y Dong PY Gurunathan S Zhang XF. Comprehensive review on the positive and negative effects of various important regulators on male spermatogenesis and fertility. Front Nutr. 2022;9:1063510. 36726821 10.3389/fnut.2022.1063510 PMC9884832

[4] Agarwal A Srivastava A Fathima F Lodhi B. Insight into epidemiology of male infertility in central India. Int J Reprod Contraception Obstetrics Gynecol. 2022;12:215–20.

[5] Washburn RL Hibler T Kaur G Dufour JM. Sertoli cell immune regulation: a double-edged sword. Front Immunol. 2022;13:913502. 35757731 10.3389/fimmu.2022.913502 PMC9218077

[6] Liu W Du L Li J He Y Tang M. Microenvironment of spermatogonial stem cells: a key factor in the regulation of spermatogenesis. Stem Cell Res Ther. 2024;15:294. 39256786 10.1186/s13287-024-03893-z PMC11389459

[7] Shah W Khan R Shah B. The molecular mechanism of sex hormones on Sertoli cell development and proliferation. Front Endocrinol (Lausanne). 2021;12:648141. 34367061 10.3389/fendo.2021.648141 PMC8344352

[8] Zhai W Tian H Liang X. Androgen blockage impairs proliferation and function of Sertoli cells via Wee1 and Lfng. Cell Commun Signal. 2024;22:498. 39407201 10.1186/s12964-024-01875-5 PMC11481299

[9] Jiang B Yang D Peng H. Environmental toxins and reproductive health: unraveling the effects on Sertoli cells and the blood-testis barrier in animals dagger. Biol Reprod. 2024;111:977–86. 39180724 10.1093/biolre/ioae126

[10] Corpuz-Hilsabeck M Culty M. Impact of endocrine disrupting chemicals and pharmaceuticals on Sertoli cell development and functions. Front Endocrinol (Lausanne). 2023;14:1095894. 36793282 10.3389/fendo.2023.1095894 PMC9922725

[11] Gao Y Wang Z Long Y. Unveiling the roles of Sertoli cells lineage differentiation in reproductive development and disorders: a review. Front Endocrinol (Lausanne). 2024;15:1357594. 38699384 10.3389/fendo.2024.1357594 PMC11063913

[12] Wen L Yuan Q Sun M. Generation and characteristics of human Sertoli cell line immortalized by overexpression of human telomerase. Oncotarget. 2017;8:16553–70. 28152522 10.18632/oncotarget.14985 PMC5369984

[13] Washburn RL Dufour JM. Complementing testicular immune regulation: the relationship between Sertoli cells, complement, and the immune response. Int J Mol Sci. 2023;24:3371. 36834786 10.3390/ijms24043371 PMC9965741

[14] Xiao Z Liang J Huang R. Inhibition of miR-143-3p restores blood-testis barrier function and ameliorates Sertoli cell senescence. Cells. 2024;13:313. 38391926 10.3390/cells13040313 PMC10887369

[15] Grogg J Schneider K Bode PK. Sertoli cell tumors of the testes: systematic literature review and meta-analysis of outcomes in 435 patients. Oncologist. 2020;25:585–90. 32043680 10.1634/theoncologist.2019-0692 PMC7356704

[16] Griswold MD. 50 years of spermatogenesis: Sertoli cells and their interactions with germ cells. Biol Reprod. 2018;99:87–100. 29462262 10.1093/biolre/ioy027 PMC7328471

[17] Li H Spade DJ. REPRODUCTIVE TOXICOLOGY: Environmental exposures, fetal testis development and function: phthalates and beyond. Reproduction. 2021;162:F147–67. 34314370 10.1530/REP-20-0592 PMC8497445

[18] Xiao Y Zhang J Guan Y. Research progress on Sertoli cell secretion during spermatogenesis. Front Endocrinol (Lausanne). 2024;15:1456410. 39882265 10.3389/fendo.2024.1456410 PMC11775901

[19] Ju F. Mapping the knowledge structure of image recognition in cultural heritage: a scientometric analysis using CiteSpace, VOSviewer, and Bibliometrix. J Imaging. 2024;10:272. 39590736 10.3390/jimaging10110272 PMC11596010

[20] Xu J Yu Y Li S Qiu F. Global trends in research of amino acid metabolism in T lymphocytes in recent 15 years: a bibliometric analysis. J Immunol Res. 2025;2025:3393342. 39950085 10.1155/jimr/3393342 PMC11824865

[21] Zhang X Ye X Xie Y. GEV (Sod2) powder: a modified product based on biovesicles functioned in air pollution PM2.5-induced cardiopulmonary injury. Research (Wash D C). 2025;8:0609. 39949511 10.34133/research.0609 PMC11822167

[22] Yu DG Xu L. Impact evaluations of articles in current drug delivery based on web of science. Curr Drug Deliv. 2024;21:360–7. 37157193 10.2174/1567201820666230508115356

[23] Wang X Cao X Dai Z Dai Z. Bibliometric analysis and visualisation of research hotspots and frontiers on omics in osteosarcoma. J Cancer Res Clin Oncol. 2024;150:393. 39172141 10.1007/s00432-024-05898-w PMC11341630

[24] Kushairi N Ahmi A. Flipped classroom in the second decade of the Millenia: a Bibliometrics analysis with Lotka’s law. Educ Inf Technol (Dordr). 2021;26:4401–31. 33686330 10.1007/s10639-021-10457-8 PMC7929902

[25] Ai Y Sun K Hu H. Bibliometric analysis of papers on mild cognitive impairment nursing in China. Int J Nurs Sci. 2017;4:73–9. 31406722 10.1016/j.ijnss.2016.10.005 PMC6626079

[26] Mruk DD Cheng CY. Sertoli-Sertoli and Sertoli-germ cell interactions and their significance in germ cell movement in the seminiferous epithelium during spermatogenesis. Endocr Rev. 2004;25:747–806. 15466940 10.1210/er.2003-0022

[27] Cheng CY Mruk DD. Cell junction dynamics in the testis: Sertoli-germ cell interactions and male contraceptive development. Physiol Rev. 2002;82:825–74. 12270945 10.1152/physrev.00009.2002

[28] Arato I Grande G Barrachina F. “In vitro” effect of different follicle-stimulating hormone preparations on Sertoli cells: toward a personalized treatment for male infertility. Front Endocrinol (Lausanne). 2020;11:401. 32625170 10.3389/fendo.2020.00401 PMC7314925

[29] Dong S Chen C Zhang J Gao Y Zeng X Zhang X. Testicular aging, male fertility and beyond. Front Endocrinol (Lausanne). 2022;13:1012119. 36313743 10.3389/fendo.2022.1012119 PMC9606211

[30] Adamczewska D Slowikowska-Hilczer J Walczak-Jedrzejowska R. The fate of leydig cells in men with spermatogenic failure. Life (Basel). 2022;12:570. 35455061 10.3390/life12040570 PMC9028943

[31] Yang T Tang D Zhan Y Seyler BC Li F Zhou B. SARS-CoV-2 vaccination and semen quality: a study based on sperm donor candidate data in southwest China. Transl Androl Urol. 2024;13:80–90. 38404555 10.21037/tau-23-395 PMC10891393

[32] Vollset SE Goren E Yuan CW. Fertility, mortality, migration, and population scenarios for 195 countries and territories from 2017 to 2100: a forecasting analysis for the global burden of disease study. Lancet. 2020;396:1285–306. 32679112 10.1016/S0140-6736(20)30677-2 PMC7561721

[33] Wei L Zhang J Deng X. Impacts of the COVID-19 pandemic on Chinese assisted reproductive technology institutions and human sperm banks: reflections in the post-pandemic era. J Health Popul Nutr. 2023;42:82. 37592335 10.1186/s41043-023-00422-1 PMC10436387

[34] Liu M He Q Yuan Z. HDAC3 promotes Sertoli cell maturation and maintains the blood-testis barrier dynamics. FASEB J. 2024;38:e23526. 38430456 10.1096/fj.202301349RR

[35] Ge X He Z Cao C. Protein palmitoylation-mediated palmitic acid sensing causes blood-testis barrier damage via inducing ER stress. Redox Biol. 2022;54:102380. 35803125 10.1016/j.redox.2022.102380 PMC9287734

[36] Wang X Liu X Qu M Li H. Sertoli cell-only syndrome: advances, challenges, and perspectives in genetics and mechanisms. Cell Mol Life Sci. 2023;80:67. 36814036 10.1007/s00018-023-04723-w PMC11072804

[37] Meng X Lindahl M Hyvönen ME. Regulation of cell fate decision of undifferentiated spermatogonia by GDNF. Science. 2000;287:1489–93. 10688798 10.1126/science.287.5457.1489

[38] Lopez-Botella A Velasco I Acien M. Impact of heavy metals on human male fertility-an overview. Antioxidants (Basel). 2021;10:1473. 34573104 10.3390/antiox10091473 PMC8468047

[39] Wei Y Zhou Y Long C. Polystyrene microplastics disrupt the blood-testis barrier integrity through ROS-Mediated imbalance of mTORC1 and mTORC2. Environ Pollut. 2021;289:117904. 34371264 10.1016/j.envpol.2021.117904

[40] Meroni SB Galardo MN Rindone G Gorga A Riera MF Cigorraga SB. Molecular mechanisms and signaling pathways involved in Sertoli cell proliferation. Front Endocrinol (Lausanne). 2019;10:224. 31040821 10.3389/fendo.2019.00224 PMC6476933

[41] Ma Q You X Zhu K. Changes in the tight junctions of the testis during aging: role of the p38 MAPK/MMP9 pathway and autophagy in Sertoli cells. Exp Gerontol. 2022;161:111729. 35134475 10.1016/j.exger.2022.111729

[42] Chen KQ Wei BH Hao SL Yang WX. The PI3K/AKT signaling pathway: how does it regulate development of Sertoli cells and spermatogenic cells? Histol Histopathol. 2022;37:621–36. 35388905 10.14670/HH-18-457

[43] Keshtmand Z Eftekhari S Khodadadi B. Engineering of gelatin scaffold by extracellular matrix of Sertoli cells for embryonic stem cell proliferation. Toxicol In Vitro. 2024;100:105900. 39029600 10.1016/j.tiv.2024.105900

[44] Cortez J Torres CG Parraguez VH De Los Reyes M Peralta OA. Bovine adipose tissue-derived mesenchymal stem cells self-assemble with testicular cells and integrates and modifies the structure of a testicular organoids. Theriogenology. 2024;215:259–71. 38103403 10.1016/j.theriogenology.2023.12.013

[45] Hashemi Karoii D Javadzadeh G Azizi H Al-Joborae FFM Amirian M. Deciphering the Sertoli cell signaling pathway with protein-protein interaction, single-cell sequencing, and gene ontology. Cell Reprogram. 2024;26:135–45. 39475771 10.1089/cell.2024.0059

[46] Falvo S Grillo G Latino D Chieffi Baccari G Di Fiore MM Venditti M. Potential role of mitochondria and endoplasmic reticulum in the response elicited by D-aspartate in TM4 Sertoli cells. Front Cell Dev Biol. 2024;12:1438231. 39105170 10.3389/fcell.2024.1438231 PMC11298366

[47] Justin Margret J Jain SK. The protective role of L-cysteine in the regulation of blood-testis barrier functions-a brief review. Genes (Basel). 2024;15:1201. 39336792 10.3390/genes15091201 PMC11430845

[48] XueXia L YaNan L Zi T. Di-2-ethylhexyl phthalate (DEHP) exposure induces sperm quality and functional defects in mice. Chemosphere. 2023;312(Pt 1):137216. 36372335 10.1016/j.chemosphere.2022.137216

[49] Wang Z Xu X. scRNA-seq profiling of human testes reveals the presence of the ACE2 receptor, a target for SARS-CoV-2 infection in spermatogonia, leydig and Sertoli cells. Cells. 2020;9:920. 32283711 10.3390/cells9040920 PMC7226809

[50] Ma X Guan C Chen R. Pathological and molecular examinations of postmortem testis biopsies reveal SARS-CoV-2 infection in the testis and spermatogenesis damage in COVID-19 patients. Cell Mol Immunol. 2021;18:487–9. 33318629 10.1038/s41423-020-00604-5 PMC7734388

[51] Duarte-Neto AN Teixeira TA Caldini EG. Testicular pathology in fatal COVID-19: a descriptive autopsy study. Andrology. 2022;10:13–23. 34196475 10.1111/andr.13073 PMC8444746

[52] Cannarella R Marino M Crafa A. Impact of COVID-19 on testicular function: a systematic review and meta-analysis. Endocrine. 2024;85:44–66. 38345682 10.1007/s12020-024-03705-7 PMC11246276

[53] Wen L Tian H Huang X. Effect of SARS-CoV-2 on semen parameters: a meta-analysis of 39 articles from 15 countries. J Global Health. 2024;14:05021. 10.7189/jogh.14.05021 PMC11364090 39212663

[54] Gan L Huang S Hu Y Zhang J Wang X. Heat treatment reduced the expression of miR-7-5p to facilitate insulin-stimulated lactate secretion by targeting IRS2 in boar Sertoli cells. Theriogenology. 2022;180:161–70. 34973648 10.1016/j.theriogenology.2021.12.029

[55] Mondal S Bandyopadhyay A. Glucose transporters (GLUTs): Underreported yet crucial molecules in unraveling testicular toxicity. Biochimie. 2024;219:55–62. 37967737 10.1016/j.biochi.2023.11.004

[56] O’Donnell L Smith LB Rebourcet D. Sertoli cells as key drivers of testis function. Semin Cell Dev Biol. 2022;121:2–9. 34229950 10.1016/j.semcdb.2021.06.016

[57] Almamoun R Pierozan P Karlsson O. Mechanistic screening of reproductive toxicity in a novel 3D testicular co-culture model shows significant impairments following exposure to low-dibutyl phthalate concentrations. Arch Toxicol. 2024;98:2695–709. 38769170 10.1007/s00204-024-03767-6 PMC11272729

[58] Wang JM Li ZF Yang WX. What does androgen receptor signaling pathway in Sertoli cells during normal spermatogenesis tell us? Front Endocrinol (Lausanne). 2022;13:838858. 35282467 10.3389/fendo.2022.838858 PMC8908322

[59] Zhang SX Wang DL Qi JJ. Chlorogenic acid ameliorates the heat stress-induced impairment of porcine Sertoli cells by suppressing oxidative stress and apoptosis. Theriogenology. 2024;214:148–56. 37875054 10.1016/j.theriogenology.2023.10.018

[60] Cannarella R Mancuso F Arato I. Sperm-carried IGF2 downregulated the expression of mitogens produced by Sertoli cells: a paracrine mechanism for regulating spermatogenesis? Front Endocrinol (Lausanne). 2022;13:1010796. 36523595 10.3389/fendo.2022.1010796 PMC9744929

